# New insights on the reorganization of gene transcription in *Pseudomonas putida* KT2440 at elevated pressure

**DOI:** 10.1186/1475-2859-12-30

**Published:** 2013-03-28

**Authors:** Stéphanie Follonier, Isabel F Escapa, Pilar M Fonseca, Bernhard Henes, Sven Panke, Manfred Zinn, María Auxiliadora Prieto

**Affiliations:** 1 Laboratory for Biomaterials, Empa (Swiss Federal Laboratories for Materials Science and Technology), St. Gallen, Switzerland; 2 Current address: Institute of Life Technologies, HES-SO Valais Wallis (University of Applied Sciences Western Switzerland), Sion, Switzerland; 3 Environmental Biology Department, Centro de Investigaciones Biológicas (CIB), Consejo Superior de Investigaciones Científicas (CSIC), Madrid, Spain; 4 Current address: Department of Microbiology, The Forsyth Institute, Cambridge, USA; 5 Department for Biosystems Science and Engineering, Bioprocess Laboratory, ETH Zurich, Basel, Switzerland

**Keywords:** *Pseudomonas putida* KT2440, Gene transcription, DNA microarrays, Elevated pressure, Dissolved oxygen tension, Environmental stress, mcl-PHA

## Abstract

**Background:**

Elevated pressure, elevated oxygen tension (DOT) and elevated carbon dioxide tension (DCT) are readily encountered at the bottom of large industrial bioreactors and during bioprocesses where pressure is applied for enhancing the oxygen transfer. Yet information about their effect on bacteria and on the gene expression thereof is scarce. To shed light on the cellular functions affected by these specific environmental conditions, the transcriptome of *Pseudomonas putida* KT2440, a bacterium of great relevance for the production of medium-chain-length polyhydroxyalkanoates, was thoroughly investigated using DNA microarrays.

**Results:**

Very well defined chemostat cultivations were carried out with *P. putida* to produce high quality RNA samples and ensure that differential gene expression was caused exclusively by changes of pressure, DOT and/or DCT. Cellular stress was detected at 7 bar and elevated DCT in the form of heat shock and oxidative stress-like responses, and indicators of cell envelope perturbations were identified as well.

Globally, gene transcription was not considerably altered when DOT was increased from 40 ± 5 to 235 ± 20% at 7 bar and elevated DCT. Nevertheless, differential transcription was observed for a few genes linked to iron-sulfur cluster assembly, terminal oxidases, glutamate metabolism and arginine deiminase pathway, which shows their particular sensitivity to variations of DOT.

**Conclusions:**

This study provides a comprehensive overview on the changes occurring in the transcriptome of *P. putida* upon mild variations of pressure, DOT and DCT. Interestingly, whereas the changes of gene transcription were widespread, the cell physiology was hardly affected, which illustrates how efficient reorganization of the gene transcription is for dealing with environmental changes that may otherwise be harmful. Several particularly sensitive cellular functions were identified, which will certainly contribute to the understanding of the mechanisms involved in stress sensing/response and to finding ways of enhancing the stress tolerance of microorganisms.

## Background

Among the various methods developed for optimizing bacterial bioprocesses, the application of elevated pressure in the range of 1–10 bar constitutes a simple and effective way to increase the oxygen transfer rate and achieve high cell densities [1-10]. In particular, this method has proved suitable for the production of medium-chain-length polyhydroxyalkanoates (mcl-PHA), biocompatible and biodegradable polymers with a wide range of applications [11-13], by *Pseudomonas putida* KT2440. Indeed, we demonstrated in a recent work that except for a small decrease of viability, the physiology of *P. putida* KT2440 (biomass production, nutrient yields, respiratory quotient, mcl-PHA production) was not impaired at 7 bar [2]. Nevertheless, pressure may still induce stress and affect metabolic pathways, cellular machineries and cellular functions when varying from optimal values, in a similar way as deviations of temperature, pH, osmolarity or water availability. Unlike for the latter environmental factors [14-16], the effect of elevated pressure (1–10 bars) on the transcriptome of bacteria, and *P. putida* KT2440 in particular, has never been investigated in detail.

Bacteria live in nature mostly at a pressure of around 1 bar with the exception of microorganisms found in the sea (pressure increase of about 0.1 bar/m), in the soil (pressure increase of about 0.3 bar/m) or in plant cells (pressures below 1 bar made possible by turgor) [17]. In general, bacteria are able to grow up to at least 300 bar but they cannot survive above 2000 bar [18]. As a result, pressure-based preserving and sterilizing methods have been developed and successfully exploited in the food industry [19] and are now being studied as alternative to heat treatment and gamma ray irradiation for the disinfection of biomaterials [20-25]. Essential processes such as DNA replication, RNA transcription and protein synthesis are functional in *Escherichia coli* up to at least 500, 200 and 600 bar, respectively [26]. Starting from 1000–2000 bar protein aggregation and nucleoid changes can be observed [27] along with loss of membrane integrity [28]. Filamentous growth [29] and loss of motility [30] are two other phenotypes of cells confronted to high-pressure stress. In addition, the composition of fatty acids in the cell membrane varies at high pressure [31] as well as the production of several membrane proteins such as OmpH and OmpL [32,33], transporters [34] and terminal oxidases [35]. Lastly, induction of heat-shock, cold-shock, and SOS responses was observed following high pressure treatment in *E. coli*[33,34,36].

Notably, all the effects described above were caused by pressures at least 20 times larger than the ones intended to be applied during bacterial fermentations. Therefore, differences are expected between the transcriptomes of cells subjected to high pressure (> 200 bar) and elevated pressure (< 10 bar). Another important point to consider under elevated pressure is the increase of dissolved oxygen tension (DOT) and dissolved carbon dioxide tension (DCT) resulting from the larger gas solubility. This phenomenon is hardly observed for processes at high pressure since they are normally performed in degassed systems. The occurrence of large DOT during a bioprocess can be avoided by decreasing the aeration or by diluting the aeration gas with nitrogen. In contrast it is much more difficult to maintain DCT values below a certain level, especially in the case of medium- or high-cell-density cultivations that produce significant amounts of CO_2_. As a result changes in DCT, and in DOT if uncontrolled, can also affect gene transcription at elevated pressure.

In order to unravel the cellular mechanisms used by *P. putida* KT2440 for sensing and responding to variations of pressure, DOT and DCT, we explored its transcriptome by means of DNA microarrays. Not only was the effect of elevated pressure and DCT studied but also the effect of combined elevated pressure, DCT and DOT. This latter effect was investigated because: (1) such conditions can readily occur if pressure is not applied exactly in parallel with the oxygen demand and (2) the effect of oxidative stress on the gene expression of *P. putida* KT2440 has not been studied yet.

Here, we report the most relevant cellular mechanisms affected by elevated pressure, DCT and DOT and provide general information for a further, in greater depth investigation of specific genes and cellular functions. In addition to extending the global understanding of stress sensing and stress response mechanisms, this study aims at identifying candidate genes that could be engineered to enhance the resistance and/or productivity of microorganisms used in industrial bioprocesses where such stresses can occur (e. g. mcl-PHA production processes at elevated pressure).

## Results

Cultivations with *P. putida* KT2440 were carried out at 1 bar (scenario “*Control”*), at 7 bar with a similar DOT as at 1 bar (“*Pressure”*), and at 7 bar with a higher DOT (“*Pressure Oxygen”*) (Table 1). Detailed description of these cultivations is reported in a previous work where the effect of pressure on cell physiology was investigated [2]. In the present work we focus on the effect of pressure on the cell transcriptome while using the same biomass samples. These biomass samples were produced in chemostat cultivations where the growth rate, cell density, medium composition, pH, and temperature were precisely controlled in order to avoid any unwanted effect on the gene transcription. The level of dissolved CO_2_ (CO_2,aq_ and HCO_3_^-^) increased from 10 mM at 1 bar to ~20 mM at 7 bar (Table 1). As a result, the changes of gene expression reported for 7 bar may be a consequence of either elevated pressure *per se*, elevated DCT, or the combination of both factors. The most relevant genes and functions affected in the *Pressure* and *Pressure Oxygen* conditions are listed in Table 2.

**Table 1:** **Characteristics of the chemostat cultivation conditions with *P. putida *KT2440 (dilution rate = 0.15 h**^**-1**^**)**

	**Control**	**Condition 1**	**Condition 2**
**Effect investigated**	**-**	***Pressure***	***Pressure Oxygen***
P [bar]	1	7	7
DOT [%]	40 ± 5	45 ± 20	235 ± 20
[CO_2_]_tot_ [mM]*	10 ± 1	21 ± 5	19 ± 2
CDW [g L^-1^]^#^	14.0 ± 0.5	14.1 ± 0.3	13.6 ± 0.8

For more details about the cultivation and sampling procedure, please refer to [2].

* Total concentration of dissolved CO_2_. ^#^ Cell dry weight.

**Table 2:** Overview of the most relevant cellular functions affected by elevated pressure alone and by elevated pressure and DOT

**Cellular function**	**Differentially expressed genes**	
	***Pressure***	***Pressure oxygen***
Stress sensing/stress response	*• aer-1* (-), MCP (-)	*• aer-1* (-), MCP (-)
	*• rpoH* and heat-shock genes (+)	*• rpoH* and heat-shock genes (+)
	*•* DNA repair (-)	*•* DNA repair (-)
Control of reactive oxygen species	*• ahpC*, glutathione genes (+)	*• ahpC*, glutathione genes (+)
	*• anr* (+) and cyo (+)	*• anr* (-) and cyo (+)
	*•* Cbb_3_-1 (+), Cbb_3_-2 (-), Aa_3_ (-), Cyo (+), *azu* (+)	*•* Cbb_3_-1 (-), Cbb_3_-2 (-), Aa_3_ (-), Cyo (+), *azu* (-)
Cell envelope	*• oprG* (+), *oprH* (+)	*• oprG* (-), *oprH* (+)
	*•* membrane proteins	*•* membrane proteins
	*•* secretion systems (+)	*•* secretion systems (+)
Internal pH	*•* urease (+)	*•* secretion systems (+)
	*•* secretion systems (+)	
	*•* oxidative stress (+)	*•* oxidative stress (+)
Fe homeostasis/Fe-S clusters	*•* siderophore transporters (+), bacterioferritin (-)	
		*•* siderophore transporters (+), bacterioferritin (-)
Biosynthetic metabolism		*•* ADI (-)
		*•* Glu synthesis/metabolism (+)

Induction and repression of gene transcription is indicated by the signs (+) and (-), respectively, and major differences of gene transcription between the two conditions are indicated by the bold font. Abbreviations: *ADI*, arginine deiminase pathway; *Glu*, glutamate; *MCP*, methyl-accepting chemotaxis protein.

### Global analyses of *P. putida* KT2440 transcriptome under elevated pressure and under combined elevated pressure and elevated DOT

We showed previously that the physiology of *P. putida* KT2440 was hardly affected at 7 bar [2]. No decrease of cell growth rate and PHA-free biomass (total cell dry weight minus PHA weight) production was observed and the respiratory quotient did not vary under these conditions, either. Nevertheless, small changes were observed in the production of mcl-PHA (increase) and in cell viability (decrease). The physiology of cells cultivated in the *Pressure Oxygen* condition was very similar, with the exception of a small decrease of respiratory quotient and slightly larger changes of polymer production and cell viability.

**Figure 1:**
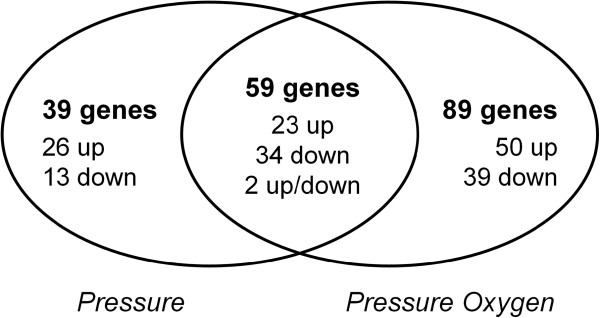
**Number of significantly differentially expressed genes.** “*Pressure*” and “*Pressure Oxygen*” refer to the genes differentially expressed at elevated pressure and at combined elevated pressure and elevated DOT, respectively. The number of genes overexpressed compared to the *Control* condition is described as “up”, the number of genes underexpressed as “down”, and the number of genes overexpressed in one condition but underexpressed in the other one as “up/down”.

In contrast to these few changes of cell physiology, increasing the pressure from 1 to 7 bar caused extensive changes of gene transcription. The DNA microarray experiments revealed significant differential expression (adjusted p-value ≤ 0.01) for about 100 and 150 genes in the *Pressure* condition and the *Pressure Oxygen* condition, respectively (Figure 1, Additional file 1: Table S1-S2). Almost 60 genes were significantly differentially expressed regardless of the DOT level but interestingly, two of them exhibited opposite differential expression (Figure 1, Additional file 1: Table S1). These two genes were coding for the outer membrane porin OprG (PP_0504) and for a hypothetical protein (PP_5390).

**Figure 2:**
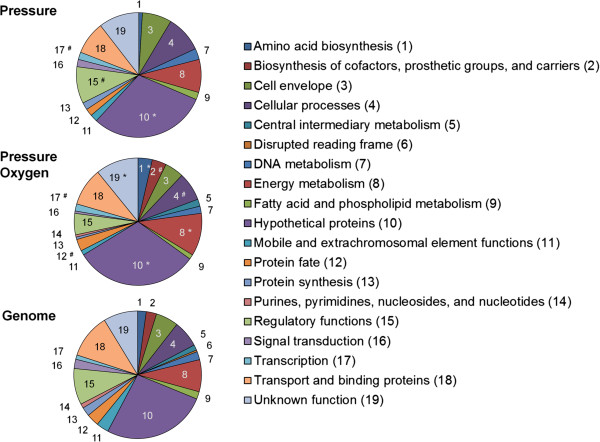
**Classification of the significantly differentially expressed genes into role categories.** The genes were sorted into the 19 role categories established by the Comprehensive Microbial Resource (CMR). “*Pressure*” and “*Pressure Oxygen*” refer to the genes differentially expressed at elevated pressure and at combined elevated pressure and elevated DOT, respectively. For comparison, the gene distribution of the complete *P. putida* KT2440 genome is also depicted (*Genome*). For clarity reasons, a number was assigned to each category and is shown in the corresponding sector of the charts. Categories that were significantly over-represented as well as categories containing sub-categories that were significantly over-represented compared to the genome distribution are marked by the asterisk (*) and the hash (#), respectively (for more details, see the Additional file 1: Table S2-S3).

The significantly differentially expressed genes were classified into categories and sub-categories according to the Comprehensive Microbial Resource (CMR) [37]. The gene repartition for the *Pressure* condition was very similar to the repartition considering the entire genome (Figure 2, Additional file 1: Table S2). In contrast, several categories were over-represented in the *Pressure Oxygen* condition compared to the genome: genes involved in the biosynthesis of amino acids such as glutamate; in the biosynthesis of cofactors, prosthetic groups, and carriers such as glutathione; in adaptation to atypical conditions; in energy metabolism; in protein folding and stabilization; and, like for *Pressure*, genes coding for hypothetical proteins and involved in transcription (Additional file 1: Table S3).

Genes coding for sensing proteins (e. g. methyl-accepting chemotaxis transducer PP_2643) and for proteins located in the cell envelope (e. g. outer membrane proteins OprG and OprH, putative multidrug efflux transporter PP_0906, members of a type VI secretion system PP_3094 and PP_3100) exhibited the highest significant differential expression in both the *Pressure* and the *Pressure Oxygen* scenarios (Additional file 1: Table S4). In addition, differential expression of genes coding for a bacterioferritin (PP_1082), for the alkyl hydroperoxide reductase AhpC (PP_2439) involved in detoxification, and for the iron-cluster assembly proteins IscS and IscA (PP_0842 and PP_0844) had a particularly high significance in the *Pressure Oxygen* condition.

In general, the transcriptomic profiles (Table 3) were supported by qRT-PCR data since out of nine genes tested, eight exhibited similar fold changes with the two methods (Table 4). For the one gene showing discrepant results (*aer-1*, PP_2257) a highly specific hydrolysis probe base real time PCR had to be performed to establish the down-regulation of the gene in the *Pressure* and *Pressure Oxygen* conditions (see the section *Methods*). At this stage, it is inherently impossible to produce DNA microarray data with neither false positives nor false negatives because of the huge amount of genes (> 5’000) tested simultaneously. As a result, follow-up studies focusing on particular genes should include confirmation experiments such as RT-PCR or production of deletion mutants.

**Table 3:** **Most relevant genes differentially expressed under elevated pressure ( *Pressure *) and under elevated pressure and DOT ( *Pressure oxygen *)**

**ID**	**Name**	**Gene**	***Pressure***		***Pressure oxygen***	
			**FC**	**Adj. P-val**	**FC**	**Adj. P-val**
Transcription regulators						
PP_2088	RNA polymerase sigma factor SigX	*sigX*	-1.88	1.9 E-03	-1.65	5.6 E-03
PP_5108	RNA polymerase sigma-32 factor	*rpoH*	+1.49*	3.6 E-03	+1.57	8.7 E-03
PP_1429	sigma E regulatory protein, MucB/RseB	*algN*	+1.64	1.5 E-03	+1.46*	1.3 E-02
PP_1863	transcriptional regulator LysR family		+2.77	3.1 E-03		
PP_2475	transcriptional regulator TetR family		+1.59	7.8 E-03	+1.75	7.5 E-03
PP_3439	transcriptional regulator AraC family		-1.55	7.7 E-03	-1.59	5.4 E-03
PP_4508	transcriptional regulator AraC family		-1.45*	1.2 E-02	-1.55	3.6 E-03
Global regulatory proteins						
PP_4265	transcriptional regulator Anr	*anr*	+1.61	1.2 E-02	-1.99	2.9 E-02
Signal sensing and transduction						
PP_2258	sensory box protein		-1.51	1.6 E-02	-1.56	3.2 E-02
PP_2643	methyl-accepting chemotaxis transducer		-6.82	2.9 E-07	-15.45	8.5 E-08
PP_1761	sensory box protein/GGDEF family protein		-1.77	1.9 E-03	-1.71	2.8 E-03
PP_1762	conserved hypothetical protein		-1.45*	2.9 E-02	-1.54	9.3 E-03
PP_2097	sensory box protein		-1.49*	5.0 E-03	-1.52	2.0 E-02
PP_4405	sensory box protein		+2.04	8.0 E-03	+1.39*	8.9 E-02
PP_5324	response regulator		-1.53	4.3 E-03	-1.47*	9.2 E-03
PP_2356	phytochrome family protein (two-component sensor activity)		-1.73	4.6 E-03	-1.85	1.3 E-03
PP_4362	Hpt protein (two-component system)		-1.36*	7.4 E-02	-1.52	9.0 E-03
PP_4503	winged helix family two component transcriptional regulator		+1.56	2.3 E-03	+1.39*	8.9 E-02
PP_0997	sigma-54 dependent transcriptional regulator/sensory box protein		-1.46	2.0 E-02	-1.88	4.7 E-04
Stress response (Chaperones, DNA repair)						
PP_4179	heat shock protein HtpG	*htpG*	+1.65	1.0 E-02	+1.92	2.0 E-02
PP_4728	heat shock protein GrpE	*grpE*	+1.33*	6.2 E-02	+1.72	7.2 E-03
PP_1360	co-chaperonin GroES	*groES*	+1.61	2.0 E-02	+1.77	8.2 E-02
PP_1361	chaperonin 60 kDa	*groEL*	+1.78	1.0 E-02	+2.19	6.0 E-03
PP_1522	cold shock protein CspA	*cspA-1*	-1.51	9.1 E-03	-1.75	3.3 E-03
PP_1092	endonuclease III	*nth*	-1.67	1.6 E-03	-1.58	3.7 E-03
PP_1624	group II intron-encoding maturase		-1.68	1.3 E-03	-1.73	1.2 E-03
PP_1630	RecX protein	*recX*	+1.54	1.5 E-03	+1.53	1.6 E-02
PP_0483	excinuclease ABC A subunit	*uvrA*			-1.67	6.1 E-03
PP_2295	antirestriction protein, putative		-1.44*	9.1 E-03	-1.51	5.4 E-03
PP_2326	universal stress protein family protein		-1.51	2.6 E-02	-1.51	1.3 E-02
Cell envelope						
PP_0267	outer membrane ferric siderophore receptor putative		+1.54	1.2 E-02	+1.73	6.6 E-03
PP_0504	outer membrane protein OprG	*oprG*	+2.55	5.8 E-04	-3.39	3.1 E-04
PP_1185	outer membrane protein H1	*oprH*	+6.63	2.9 E-04	+4.76	1.1 E-04
PP_3293	conserved hypothetical protein (predicted ion channel)		-1.47*	4.5 E-03	-1.74	6.4 E-03
PP_4454	opine ABC transporter permease protein putative		+1.95	2.6 E-03	+1.36	4.6 E-02
PP_4465	porin putative		+2.33	1.9 E-03	+1.60	1.1 E-01
PP_2445	integral membrane protein TerC		-1.95	1.9 E-03	-2.25	1.3 E-03
PP_1150	membrane protein putative				-4.14	3.1 E-04
PP_4592	membrane protein putative		-1.74	3.1 E-03	-2.20	1.9 E-03
PP_0916	transporter LysE family				+1.83	1.6 E-03
PP_1068	amino acid ABC transporter ATP-binding protein				+1.80	1.3 E-03
PP_3023	amino acid efflux protein putative				+1.51	6.4 E-03
PP_5073	conserved hypothetical protein (periplasmic protein)		+5.70	1.9 E-03	+5.98	8.1 E-06
PP_4841	branched-chain amino acid ABC transporter, periplasmic amino acid-binding protein, putative		+1.95	8.5 E-03		
PP_4842	branched-chain amino acid ABC transporter, permease protein, putative		+3.07	4.9 E-03		
PP_5233	ammonium transporter	*amtB*	+1.87	8.0 E-03		
PP_4867	extracellular ligand-binding receptor		+1.79	3.3 E-03		
PP_1076	glycerol uptake facilitator protein	*glpF*	+2.23	3.0 E-03	+2.03	4.7 E-04
PP_2454	ribose ABC transporter	*rbsB*	+1.88	1.4 E-02	+3.81	5.4 E-05
PP_2119	ABC efflux tranporter/ATP-binding protein		-2.19	4.1 E-03	-2.10	1.2 E-03
PP_0508	conserved hypothetical protein (same operon as efflux ABC transporter ATP-binding prot.)		+2.55	8.8 E-03	+3.36	1.8 E-02
PP_5307	biopolymer transport protein ExbD	*exbD*			+2.30	1.8 E-03
PP_0906	multidrug efflux RND transporter putative		-2.11	1.4 E-04	-2.19	2.1 E-03
PP_0033	sugar transferase putative		-1.74	1.9 E-03	-2.20	9.6 E-03
PP_3422	lytic transglycosylase	*ltg*	-1.74	1.9 E-03	-1.97	9.9 E-04
PP_4897	N-acetylmuramoyl-L-alanine amidase		+1.67	3.6 E-03		
PP_4384	flagellar basal body L-ring protein		-1.48*	5.6 E-03		
Secretion systems						
PP_0806	surface adhesion protein putative (same operon as type I secretion system)		-1.58	6.2 E-03	-1.39	5.2 E-02
PP_1055	type II secretion pathway protein GspN	*gspN*	+1.52	3.1 E-03		
PP_3087	excinuclease ABC A subunit putative (type VI)		+1.64	3.1 E-03	+2.27	1.7 E-03
PP_3093	conserved hypothetical protein (type VI)		+1.65	6.1 E-03	+2.39	3.6 E-03
PP_3094	hypothetical protein (type VI)		+1.61	1.0 E-02	+2.60	3.5 E-05
PP_3095	chaperone-associated ATPase putative (type VI)		+1.60	2.0 E-02	+1.79	5.6 E-03
PP_3096	hypothetical protein (type VI)		+1.60	1.1 E-02	+1.97	2.4 E-02
PP_3098	conserved hypothetical protein (type VI)				+2.27	2.3 E-04
PP_3099	hypothetical protein (type VI)		+1.65	1.5 E-02	+2.00	1.1 E-02
PP_3100	conserved hypothetical protein (type VI)		+2.25	2.9 E-04	+2.69	9.6 E-04
PP_3782	hypothetical protein		+1.55	1.3 E-02	+1.91	7.6 E-04
PP_3783	conserved hypothetical protein		+1.60	2.7 E-03	+1.95	2.8 E-04
PP_3784	conserved domain protein				+1.78	7.7 E-03
PP_3785	hypothetical protein		+1.65	1.0 E-02	+1.87	3.2 E-03
PP_3786	aminotransferase		+1.45*	6.2 E-02	+1.78	7.3 E-03
PP_3787	hypothetical protein		+2.10	5.3 E-03	+2.35	9.2 E-04
PP_3788	non-ribosomal peptide synthetase putative		+2.35	3.7 E-03	+2.79	4.2 E-05
PP_3790	diaminopimelate epimerase	*dapF*			+1.99	1.4 E-03
Biosynthetic metabolism (amino acids - CO_2_ production/consumption - glutamate metabolism)						
PP_1143	3-hydroxyisobutyrate		-1.69	1.0 E-02	-1.95	1.8 E-03
PP_4617	leucine dehydrogenase		-1.77	1.9 E-03	-1.62	3.0 E-03
PP_4794	leucyl-tRNA synthetase	*leuS*	-1.92	3.4 E-04	-1.73	7.5 E-03
PP_0432	N-acetyl-gamma-glutamyl-phosphate reductase	*argC*			+2.03	1.7 E-03
PP_0997	sigma-54 dependent transcriptional regulator/sensory box protein		-1.46*	2.0 E-02	-1.88	4.7 E-04
PP_0999	carbamate kinase	*arcC*			-1.72	1.3 E-03
PP_1000	ornithine carbamoyltransferase	*argI*			-1.53	9.6 E-03
PP_1001	arginine deiminase	*arcA*			-1.73	1.9 E-03
PP_1002	arginine/ornithine antiporter	*arcD*			-1.66	1.2 E-02
PP_1079	arginine deiminase	*argF*			+1.69	3.9 E-03
PP_3775*	sarcosine oxidase, putative				+1.42	6.8 E-03
PP_3776	rarD protein	*rarD-3*			+1.71	3.6 E-03
PP_3777	hypothetical protein				+1.52	2.4 E-02
PP_3780	hypothetical protein		+1.44*	8.0 E-03	+1.72	1.4 E-03
PP_4594	cystathionine gamma-lyase		-1.69	1.9 E-03	-1.78	1.9 E-02
PP_1631	hypothetical protein (possible lysine decarboxylase)		-1.91	3.0 E-03	-1.62	1.3 E-02
PP_3662	decarboxylase family protein (possible lysine decarboxylase)		-1.85	4.9 E-03	-1.69	3.9 E-03
PP_1389	carboxyphosphonoenolpyruvate phosphonomutase, putative		-1.54	3.7 E-03		
PP_5346	pyruvate carboxylase subunit B	*oadA*	-1.58	1.7 E-02	-1.51	1.1 E-01
PP_5347	pyruvate carboxylase subunit A	*accC-2*	-2.23	1.9 E-03	-2.41	5.6 E-03
PP_5075	glutamate synthase subunit beta	*gltD*			+1.69	1.3 E-02
PP_5409	glucosamine--fructose-6-phosphate aminotransferase	*glmS*			+1.54	1.3 E-02
Electron transport						
PP_4265	transcriptional regulator Anr	*anr*	+1.61	1.2 E-02	-1.99	2.9 E-02
PP_0103	cytochrome c oxidase subunit II		-1.41*	1.2 E-02	-1.67	2.3 E-03
PP_0104	cytochrome c oxidase subunit I				-1.68	5.6 E-03
PP_0111	electron transport protein SCO1/SenC		-1.46*	1.3 E-02	-1.44*	8.7 E-03
PP_0811	cyoups2 protein	*cyoups2*	+1.79	1.9 E-03	+1.95	7.2 E-04
PP_0812	cytochrome o ubiquinol oxidase subunit II	*cyoA*	+1.43*	1.3 E-02	+1.59	3.0 E-03
PP_0813	cytochrome o ubiquinol oxidase, subunit I	*cyoB*			+1.41*	9.6 E-02
PP_0816	protoheme IX farnesyltransferase	*cyoE-2*			+1.41*	2.1 E-02
PP_4119	NADH dehydrogenase I A subunit	*nuoA*	-1.51	2.0 E-02	-1.66	3.6 E-03
PP_4202	electron transfer flavoprotein beta subunit		+1.42*	6.2 E-03	+1.82	1.4 E-03
PP_4250	cytochrome c oxidase cbb3-type, subunit II	*ccoN-1*	+1.51	7.2 E-02	-1.56	1.0 E-02
PP_4251	cytochrome c oxidase cbb3-type, subunit II	*ccoO-1*			-2.22	1.7 E-03
PP_4252	cytochrome c oxidase, cbb3-type, CcoQ subunit	*ccoQ-1*			-1.65	9.2 E-02
PP_4253	cytochrome c oxidase, cbb3-type, subunit III	*ccoP-1*			-1.69	2.6 E-02
PP_4255	cytochrome c oxidase cbb3-type, subunit I	*ccoN-2*	-1.39*	3.0 E-02	-1.55	7.4 E-04
PP_4870	azurin	*azu*	+2.22	1.9 E-03	-1.19	2.0 E-01
Fe-S cluster assembly						
PP_0841	iron-sulfur cluster assembly transcription factor IscR	*iscR*			+1.67	2.3 E-03
PP_0842	cysteine desulfurase	*iscS-1*			+2.50	5.7 E-05
PP_0843	iron-binding protein IscU	*iscU*			+2.48	1.5 E-04
PP_0844	iron-binding protein IscA	*iscA*			+2.19	1.1 E-04
PP_0845	co-chaperone HscB	*hscB*			+2.46	7.6 E-04
PP_0846	chaperone protein HscA	*hscA*			+1.88	1.7 E-02
PP_0847	ferredoxin, 2Fe-2S				+1.72	2.2 E-03
PP_0848	conserved hypothetical protein		-1.38	4.6 E-02	+1.52	5.2 E-03
PP_2378	yghI protein	*yghI*			+2.14	1.5 E-04
PP_1082	bacterioferritin	*bfr*	-1.85	1.3 E-02	-4.20	1.1 E-05
PP_1083	BFD domain protein (2Fe-2S)-binding domain protein				+2.35	1.4 E-04
PP_4900	iron-sulfur cluster-binding, putative		-1.43*	4.5 E-03	-1.49*	7.3 E-02
PP_5306	ferric siderophore transport system protein ExbB	*exbB*			+1.87	3.0 E-02
PP_5307	ferric siderophore transport system inner membrane protein	*exbD*			+2.30	1.8 E-03
PP_5308	TonB family protein	*tonB*			+1.54	2.4 E-02
PP_0267	outer membrane ferric siderophore receptor putative		+1.54	1.2 E-02	+1.73	6.6 E-03
Detoxification						
PP_2439	alkyl hydroxide reductase C subunit	*ahpC*	+1.87	3.8 E-03	+3.89	2.5 E-05
PP_3639	alkylhydroperoxidase		-1.68	6.7 E-03	-1.59	2.1 E-03
PP_1684	major facilitator transporter				+1.51	7.5 E-03
PP_1686	glutathione peroxidase				+2.11	3.1 E-04
PP_3444	glyoxalase/bleomycin resistance protein/dioxygenase		-1.57	4.5 E-03	-1.49*	2.9 E-02
PP_2474	glutathione S-transferase family protein		+1.69	7.2 E-02	+1.67	6.7 E-02
PP_3742	glutathione S-transferase family protein				+1.53	5.1 E-03

The genes were sorted according to their function. Differential expression is expressed in fold change (FC) where positive and negative values indicate how many times the gene transcription is increased and decreased, respectively, compared to the *Control*. The significance of differential expression is given by the adjusted P-value (Adj. P-val).

* Genes with a fold change slightly below 1.5 but whose differential expression is supported by a low adjusted P-value or by the differential expression of genes belonging to the same operon.

**Table 4:** Comparison between the differential expressions measured using DNA microarrays and qRT-PCR

			***Pressure***			***Pressure oxygen***		
			**DNA microarrays**		**qRT-PCR**	**DNA microarrays**		**qRT-PCR**
ID	Name	Gene	FC	Adj. P-val	FC	FC	Adj. P-val	FC
PP_4265	transcriptional regulator Anr	*anr*	+ 1.6	1.2 E-02	+ 1.3	- 2.0	2.9 E-02	- 2.6
PP_2257	aerotaxis receptor Aer-1	*aer-1*	+ 2.8	4.8 E-05	- 3.5	+ 2.6	1.1 E-05	- 1.7
PP_2258	sensory box protein		- 1.5	1.6 E-02	- 1.6	- 1.6	3.2 E-02	- 2.2
PP_0504	outer membrane OprG	*oprG*	+ 2.5	5.8 E-04	+ 3.9	- 3.4	3.1 E-04	- 9.4
PP_3087	excinuclease ABC A subunit putative (type VI)		+ 1.6	3.1 E-03	+ 1.4	+ 2.3	1.7 E-03	+ 1.9
PP_0104	cytochrome c oxidase subunit I		n. s.		- 2.3	- 1.7	5.6 E-03	- 4.8
PP_4255	cytochrome c oxidase cbb3-type, subunit I	*ccoN-2*	- 1.4	3.0 E-02	- 1.7	- 1.5	7.4 E-04	- 1.9
PP_4870	azurin	*azu*	+ 2.2	1.9 E-03	+ 2.0	- 1.2	2.0 E-01	- 1.9
PP_2439	alkyl hydroxide reductase C subunit	*ahpC*	+ 1.9	3.8 E-03	+ 2.1	+ 3.9	2.5 E-05	+ 3.7

Nine of the most relevant genes affected by elevated pressure (*Pressure*) and elevated pressure and dissolved oxygen (*Pressure Oxygen*) were tested. Differential expression is given in fold change (FC) where positive and negative values indicate how many times the gene transcription is increased and decreased, respectively, compared to the *Control*. The significance of differential expression is given by the adjusted P-value (Adj. P-value).

n. s. = no significant differential expression measured.

### Role of methyl-accepting chemotaxis sensory transducers PP_2643 and PP_2257 (*aer-1*) at 7 bar

The gene with the highest significance level of differential expression for both the *Pressure* and the *Pressure Oxygen* conditions was the methyl-accepting sensory transducer gene PP_2643 (down-regulated in both conditions, Additional file 1: Table S4). In addition, the aerotaxis receptor gene *aer-1* (PP_2257) and its co-transcript PP_2258 were both shown to be repressed under these two conditions by qRT-PCR (Table 4).

Bacterial aerotaxis receptors detect changes in the redox state of the electron transport system via an FAD-containing PAS (Per-Arnt-Sim) system and enable the cells to move towards more favorable environments in a similar way as for photo- or redox-taxis [38]. Aer-1 is one of the three Aer-like receptors of *P. putida* KT2440, along with Aer-2 (PP_2111) and Aer-3 (PP_4521). These three receptors are all localized at one pole on the cell and have a sequence similarity: 1. predicted PAS homology domain with signature residues for the binding of FAD; 2. hydrophobic membrane-spanning region; 3. methyl-accepting chemotaxis protein (MCP) domain [39]. Whereas a lack of Aer-2 mediates defects in metabolism-dependent taxis and aerotaxis, mutations of *aer-1* and *aer-3* genes have no reported phenotypes [39]. The gene *aer-1* forms a bicistronic operon with PP_2258 which codes for a sensory box protein and whose mutation was shown to cause motility defects in *P. putida* KT2440 [39].

Since both the DOT level and the medium composition were the same between the *Control* and the *Pressure* conditions (Table 1) they cannot be responsible for the repression of *aer-1* and PP_2258 transcription. This suggests that this aerotaxis operon may have an alternative role in sensing pressure and/or DCT, possibly via detection of changes in the redox state of the cell.

Regarding PP_2643, no information about its function or regulation is available to date. Nevertheless, since PP_2643 is a member of the MCP family one could speculate that it may work as a partner of *aer-1* and PP_2258 for sensing and/or responding to environmental changes. In agreement with it, PP_2643 transcription was significantly repressed in both the *Pressure* and *Pressure Oxygen* conditions.

### Activation of heat-shock response and repression of the expression of the cold-shock protein CspA and DNA repair proteins at 7 bar

The DNA microarray data revealed the induction of the sigma factor responsible for heat-shock response (RpoH) and of the heat-shock proteins HtpG, GrpE, GroES and GroEL at elevated pressure, i. e. both in the *Pressure* and *Pressure Oxygen* conditions (Table 3). RpoH induction is not restricted to dealing with temperature increase but plays a more general role in stress response: it can occur following DNA damage, oxidative stress, exposure to antibiotics and heavy metals, phage infection, and carbon source or amino acid starvation [40]. In addition, heat-shock induction was observed at high pressure (300 bar and above) [41-43] and heat-shock proteins were shown to provide protection against high-pressure induced damage [41].

Quite surprisingly, high pressure does not only induce heat-shock in bacteria but also cold-shock [34,43,44]. The heat-shock and cold-shock responses have been proposed to perform complementary functions, the former coping with protein denaturation/destabilization and the latter helping to maintain membrane fluidity and efficient protein synthesis [27,45-48]. In addition to these two stress responses, Aertsen *et al*. observed induction of the SOS response (*uvrA*, *recA*, and *sulA*) in *E. coli* from 750 bar, possibly caused by the disassembly of multisubunit proteins involved in DNA replication [36].

In contrast to these studies performed at high pressure, we observed a repression of the major cold-shock protein CspA (PP_1522) at moderate pressure (Table 3). Also repressed were the DNA repair genes *uvrA* and *nth* as well as the group II-intron maturase PP_1624 and the putative antirestriction protein PP_2295. However, RecX, which inhibits the DNA repair protein RecA but is co-transcribed with it [49,50], was induced in both conditions. Therefore, it seems that the DNA repair system and possibly the cold-shock response were to some extent affected for *P. putida* KT2440 at 7 bar but in the opposite way as reported for *E. coli* at much higher pressure.

### Induction of detoxification agents at elevated pressure

*P. putida* possesses several detoxification agents dealing with reactive oxygen species (ROS): superoxide dismutases (PP_0915, PP_0946), catalases (PP_0115, PP_0481, PP_2887, PP_2668), the alkylhydroperoxide reductase Ahp (PP_2439-2440, PP_3639), and other various peroxidases (PP_0777, PP_1686, PP_1859, PP_1874, PP_2943, PP_3248, PP_3587). Further antioxidant agents are thioredoxins (PP_0786, PP_5069), glutaredoxins (PP_2958, PP_5054), and glutathione reductase (PP_3819). Exclusively *ahpC* and genes related with glutathione were differentially expressed in the *Pressure* and the *Pressure Oxygen* conditions, with higher extent and significance level for the *Pressure Oxygen* condition. Induction of *ahpC* transcription in both conditions was confirmed by qRT-PCR (Table 4). Surprisingly, none of the differentially expressed genes coded for catalases or superoxide dismutases (Table 3). This suggests, first, that the increase in DOT between the *Pressure* and *Pressure Oxygen* cultivations did not generate a large production of reactive oxygen species and second, that a mild oxidative stress-like response was induced at elevated pressure. The latter statement is also supported by the differential expression of genes involved in electron transport and in iron homeostasis observed at 7 bar (see below).

### Effect of elevated pressure on the cell envelope

One of the most striking outcomes of the DNA microarray analyses was the impressive number of cell envelope proteins that were differentially regulated at elevated pressure. Amongst these were found porins, transporters, secretion system proteins, and various other membrane proteins with known or unknown functions (Table 3). In particular, the outer membrane porins OprG and OprH were induced with a very high significance level in the *Pressure* condition (Additional file 1: Table S4). OprH was strongly induced in the *Pressure Oxygen* condition as well but not OprG which was repressed, very likely because of a direct control by the oxygen sensor Anr [51]. The repression and induction of *oprG* transcription for *Pressure* and *Pressure Oxygen*, respectively, were validated by qRT-PCR (Table 4). The role of OprG porin has not been clearly established yet and contradictory results were reported about a possible function in iron and antibiotic uptake [51-54]. In contrast, OprH was shown to be regulated by the PhoP/PhoQ two-component system and overproduced under Mg^2+^-, Ca^2+^-, Mn^2+^-, and Sr^2+^-low conditions, possibly to stabilize the outer membrane by replacing the divalent cations [55,56]. The induction of these two outer membrane porins at elevated pressure might therefore be the consequence of a destabilized membrane.

A further interesting finding was the clear overexpression of two secretion systems located in the cell membrane at 7 bar: the PP_3084-3101 type VI secretion system and the PP_3775-3790 secretion system possibly involved in phytotoxicity (Table 3). Type VI secretion systems have been described for the first time in 2006 in *Vibrio cholerae* and *Pseudomonas aeruginosa*[57,58]. They are involved in the transport of proteins across the cell envelope of Gram-negative bacteria and are key virulence factors of several pathogenic bacteria [59]. Since they are also present in non-pathogenic organisms, alternative functions in host/symbiont communication, biofilm formation, quorum sensing modulation and general stress response have been proposed [60-62]. *P. putida* KT2440 possesses three such secretion systems: PP_2610-2632, PP_3084-3101, and PP_4066-4085 [61]. The second of these secretion systems was found to be induced both in the *Pressure* and the *Pressure Oxygen* conditions (results confirmed by qRT-PCR, see Table 4). Reva *et al*. showed that one gene of this secretion system (PP_3091) was repressed (and not induced) upon exposure to low temperature, urea, and benzoate stress [16]. This suggests that this type VI secretion system is sensitive to changes of environmental conditions in general. Regarding the PP_3775-3790 secretion system, it is located in a genomic island with atypical sequence features that was probably acquired from exogenous sources [63,64]. This gene cluster contains two distinct parts: ORFs PP_3781-3790 that are involved in the biosynthesis of lipodepsinonapeptide phytotoxins [65,66] and ORFs PP_3775-3780 that have been linked to amino acid biosynthesis and modification [64]. Two members of the H-NS-like MvaT family of transcriptional regulators have been reported to regulate these two gene clusters: TurA mediates the transcription of both PP_3781-3790 and PP_3775-3780 putative operons and its paralogue TurB regulates the transcription of PP_3781-3790 only [64]. In our experiments, we observed an induction of PP_3781-3790 for the *Pressure* condition and of both PP_3781-3790 and PP_3775-3780 for the *Pressure Oxygen*, which suggests that TurB and TurA, respectively, may play a role in the gene regulation under these two growth conditions.

### Effect of elevated DCT on *P. putida* transcriptome

Although not very large, the increase in the concentration of dissolved CO_2_ species [CO_2_]_tot_ between 1 bar and at 7 bar might be responsible for some of the gene expression changes observed (Table 1). Indeed, we noticed in a previous work that *P. putida* KT2440 cultivated at 1 bar with DCT levels similar as those found in the *Pressure* and *Pressure Oxygen* conditions underwent a few physiological changes (e. g. growth rate, cell yields) compared to cells cultivated at DCT levels similar to those found in the *Control* condition [2].

High DCT can (i) affect the function of biological membranes thereby interfering with cell division, substrate uptake and transport, (ii) acidify the internal pH, (iii) affect carboxylation/decarboxylation reactions, (iv) alter the physico-chemical properties of enzymes and their function, and (v) regulate virulence and toxin production in several pathogens [67-69].

Perturbations of the cell membrane and induction of virulence-like responses at elevated pressure were strongly suggested by the transcriptome analyses (see above) but these effects may not be restricted to the action of CO_2_. Only a small number of genes encoding enzymes involved in carboxylation/decarboxylation reactions (PP_1631, PP_1389, PP_3662, PP_5346/*oadA*, PP_5347/*accC-2*) were differentially regulated in the *Pressure* and *Pressure Oxygen* conditions (Table 3). For some genes, the differential expression was in agreement with an increase of DCT but for others it was not, possibly because of an additional role of these the corresponding enzymes in pH control (e. g. pyruvate carboxylases, see below).

The effect of low pH on *E. coli* transcriptome was studied by Maurer *et al.* who observed an acceleration of acid consumption and proton export as well as the coinduction of oxidative stress and heat shock regulons [70]. In addition, low pH was shown to induce the uptake of membrane permeable acids that dissipate the proton potential [71] and to repress sugar transporters and the maltose regulon in order to reduce sugar fermentation and the production of small acids [72]. In contrast, the tricarboxylic acid cycle (TCA) consumes acids and decarboxylation of amino acids (lysine, arginine) produces alkaline amines, which makes them suitable for counterbalancing pH acidification [73-75]. Also, urease, which produces two molecules of ammonia and one of CO_2_, is used by some microorganisms to increase the pH [76,77]. However, it should be noted that decarboxylases produce CO_2_ molecules which is in general unfavorable at large DCT. Lastly, acidic pH has been linked to virulence in different pathogens [78-80].

In contrast to most studies investigating the effect of low pH stress, the external pH was constantly controlled to ~ 7 during the experiments presented here. This means that if acidification of the cytoplasm occurred at elevated pressure, it was caused by an increase in the intracellular DCT and not by a flux of protons arising from the extracellular medium. As a result, differences can be expected compared with the studies mentioned above.

The DNA microarray data did not indicate a strong acidification of the cytoplasm. Therefore, the changes observed in the cell envelope and the induction of the virulence-like secretion systems at 7 bar - both of which have been linked to pH acidification and elevated DCT as mentioned above - are more likely to be explained by an elevated DCT than by internal acidification.

### Role of the oxygen sensor Anr and effect on the electron transport machinery at elevated pressure and at combined elevated pressure and DOT

Anr is the global transcriptional regulator responsible for oxygen sensing in *P. putida* and is the homologue of *E. coli* Fnr [81,82]. Its level has been reported to increase following oxygen limitation, leading to the induction of the arginine deiminase (ADI) pathway, nitrate respiration, hydrogen cyanide biosynthesis and to the overexpression of azurin and *oprG* in *P. aeruginosa*[51,82,83]. Anr also coordinates the regulation of three terminal oxidases (see below) and seems to be involved in the regulation of PHB biosynthesis genes in *Pseudomonas extremaustralis*[84]. The DNA microarray analyses we performed revealed a decrease in the transcription of *anr* for the *Pressure Oxygen* condition, which was confirmed by qRT-PCR (Table 4) and is in line with its role as oxygen sensor. Furthermore, Anr repression was supported by the differential expression of three ADI genes *arc*, *argI*, and *arcA* (PP_0999, PP_1000, and PP_1001), of azurin (*azu,* PP_4870; confirmed by qRT-PC, see Table 4), of *oprG* (PP_0504), of the Cyo genes *cyoA, cyoB* and *cyoE-2* (PP_0812, PP_0813 and PP0816), and of the cbb3-1 type genes *ccoO-1, ccoQ-1, ccoN-1 and ccoP-1* (PP_4250-4253) (Table 3). Unexpectedly, the transcription of *anr* was found to be induced in the *Pressure* condition from both DNA microarray and qRT-PCR experiments (Table 4). This suggests that Anr may play an alternative role in the sensing of pressure, carbon dioxide or stress in general.

**Figure 3:**
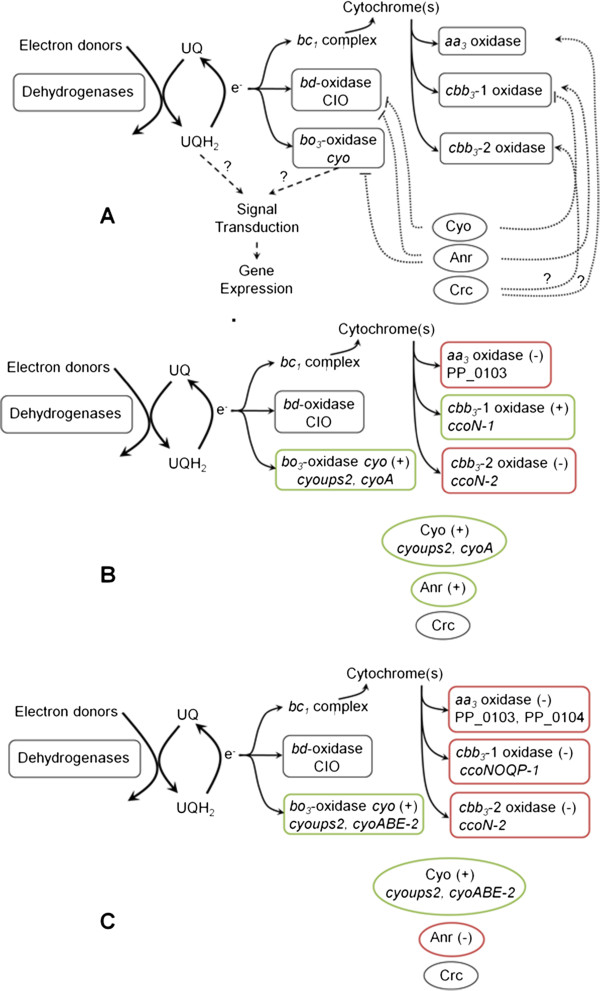
**Changes of gene transcription related to the electron transport machinery. A**. Model of *P. putida* KT2440 electron transport chain, adapted from [116]. Electrons are delivered from different electron donors to the ubiquinones located in the cell membrane (UQ). They are then transferred to the terminal ubiquinol oxidases CIO or Cyo, or are directed to the bc1 complex. In the latter case, electrons are fed to the terminal oxidases Aa_3_, Cbb_3_-1, or Cbb_3_-2 via cytochromes. The Cyo oxidase as well as the global regulators Anr and Crc are believed to modulate the expression of some of the terminal oxidase genes (dotted lines, see the text for the details). **B**. Genes coding for the five terminal oxidases and for their proposed regulators whose expression was up-regulated (+) or down-regulated (-) in the *Pressure* condition. **C**. Same as panel B but for the *Pressure Oxygen* condition.

*P. putida* KT2440 contains five different terminal oxidases (Figure 3A) with presumably different redox properties, affinity for oxygen and ability to pump protons: the cyanide-insensitive oxidase (CIO), the cytochrome o ubiquinol oxidase (Cyo), the cytochrome *aa*_*3*_ oxidase (Aa_3_), the cytochrome *cbb*_*3*_*-1* oxidase (Cbb_3_-1) and the cytochrome *cbb*_*3*_*-2* oxidase (Cbb_3_-2). Regulation of the terminal oxidases is quite complex and has not been completely unraveled to date. It involves several regulators amongst which the oxygen sensor Anr [85], the Cyo terminal oxidase [86], and the global regulator Crc (computational prediction) [87] have been identified.

Cytochrome cbb_3_-type oxidases have a very high affinity for oxygen and are therefore mostly important under oxygen-limiting conditions [88]. Expression of Cbb_3_-1 is activated by Anr [85], repressed by Cyo [86] and, together with Cbb_3_-2, possibly regulated by Crc [87]. In contrast to Cbb_3_, Cyo is predominant under high oxygen levels. It is repressed by Anr under low-oxygen conditions and during stationary phase and also acts as a global regulator [85,86,89]. CIO expression is repressed by Anr when the cells enter the stationary phase or during growth under oxygen-limiting conditions [85] and is in addition negatively regulated by Cyo [86]. Since Cyo is scarce whereas Anr is in its active form when oxygen is limiting, their effects on CIO regulation are antagonistic. As a matter of fact, the expression of CIO is induced under these conditions which means that the action of CIO is predominant [86] and it was proposed that Anr regulation is used by the cells to prevent an excessive expression of CIO compared to the other terminal oxidases [85]. Lastly, Cbb_3_-2 and Aa_3_ are neither regulated by Anr nor by Cyo [85,86] but their expression may be controlled by Crc [87].

The composition of terminal oxidases has been reported to vary with pressure in the piezophilic bacteria *Shewanella* sp. strain DB-172 F: this strain expressed cytochrome c as terminal oxidase at 1 bar but quinol oxidase at 600 bar [90]. The transcriptome analyses performed in this work revealed changes in the expression of terminal oxidases even at much more moderate pressure: the cytochrome c oxidases Aa_3_ and Cbb_3_-2 were repressed at 7 bar while the cytochrome o ubiquinol oxidase Cyo was induced (Table 3, Figure 3B and C). These differential expressions probably arose from the elevated pressure but an effect of the higher DCT or of a change in the cellular redox state cannot be excluded, either.

As expected from the higher DOT, Cbb_3_-1 (*ccoNOQP-1*) and Cbb_3_-2 (*ccoN-2*) were repressed in the *Pressure Oxygen* condition (Table 3, Figure 3C) and the repression of the latter was validated by qRT-PCR (Table 4). The down-regulation of Cbb_3_-1 is in agreement with the repression of Anr (PP_4265) and the activation of Cyo. Cyo, however, was induced both for *Pressure* and *Pressure Oxygen* (*cyoups-2, cyoA,* and *cyoups-2, cyoA, cyoB, cyoE-2*, respectively) and we observed as well a repression of Cbb_3_-2 and Aa_3_ in both cultivation conditions (confirmed by qRT-PCR, Table 4). This suggests that elevated pressure and/or DCT affected the transcription of these three terminal oxidases whereas the expression CIO remained unchanged.

### Influence of elevated DOT on iron-sulfur cluster assemblies and on iron homeostasis

Iron-sulfur clusters are ubiquitous cofactors of proteins that are involved in various cellular functions such as catalysis, electron transport, and environment sensing due to their redox properties [91]. These clusters are sensitive to oxygen and prone to decomposition if not deeply buried in the polypeptides [92]. Two different systems are responsible for the assembly and delivery of Fe-S clusters in *E. coli*: the ISC (iron-sulfur cluster) system, which functions under normal growth conditions and is inactivated under oxidative stress, and the SUF (sulfur assimilation) system, which is induced under oxidative stress and iron scarcity [91]. Only the ISC system is present in *P. putida* KT2440, which suggests that it is more resistant than the *E. coli* equivalent or that the strain possesses additional systems, not described so far, able to take over under oxidative stress. Expression of the ISC genes was clearly up-regulated in the *Pressure Oxygen* condition (PP_0841-0848; Table 3). It should be noted that this does not necessarily imply that the ISC machinery was more effective since Jang and Imlay showed that although the expression of ISC genes was induced by H_2_O_2_ stress in *E. coli* the system was inactive [93].

In addition to the ISC genes, the transcription of *yhgI* (PP_2378, also known as *gntY* or *nfuA*) was induced in the *Pressure Oxygen* condition. NfuA is involved in the biogenesis of Fe-S clusters in *E. coli* and *Azobacter vinelandii*[94,95] and may play a general role in the repair of damaged Fe-S proteins under stress conditions [94]. The *nfuA* gene was shown to belong to the *iscR* and *rpoH* regulons in *E. coli*[96,97]. Nevertheless, *rpoH* regulation seems to be less strict in *P. putida* since its overexpression did not result in the induction of NfuA/YhgI in the *Pressure* condition.

Not only the Fe-S clusters are sensitive to oxygen but iron itself. Indeed, Fe^2+^, which is soluble at physiological pH, can be oxidized into Fe^3+^ which readily precipitates as ferric hydroxide or forms insoluble complexes with anionic salts. Bacteria are endowed with ferritin and bacterioferritin proteins (without and with heme, respectively) that control the intracellular level of iron between ~10^-3^-10^-5^ M: they store excess iron in order to prevent the formation of ROS formation via Fenton reactions and release it according to the cellular needs [98,99]. We observed a significant repression of transcription for the bacterioferritin gene *bfr* (PP_1082) in the *Pressure Oxygen* condition but induction of the putative bacterioferritin-associated ferredoxin (PP_1083) that is located next to it in *P. putida* genome. Interestingly, Tuanyok and coworkers detected a similar regulation in *Burkholderia pseudomallei* and *Burkholderia mallei* when cultivated under low iron conditions [100]. This suggests that the putative bacterioferritin-associated ferredoxin may be involved in the release of iron from bacterioferritin and that the level of free iron was lower in the *Pressure Oxygen* condition than in the *Control*. Lastly, it must be noted that the expression of *bfr* and of two siderophore transporter genes was differentially regulated in the *Pressure* condition (Table 3). We propose that this apparent perturbation of iron homeostasis at 7 bar was the consequence of a mild oxidative stress.

### Induction of glutamate metabolism and repression of the arginine deiminase pathway at elevated DOT

The global analyses of the transcriptome data suggested an effect of elevated DOT on glutamate biosynthesis (see above). Examination of the genes involved in glutamate metabolism [101] revealed that a glutamate synthase (PP_5075) and a gene involved in the metabolization of glutamine (PP_5409) were induced in the *Pressure Oxygen* condition (Table 3). The enhancement of glutamate synthesis and metabolism at elevated DOT may be explained by an acceleration of the tricarboxylic acid cycle (TCA) triggered by larger NAD^+^/NADH and FAD/FADH_2_ ratios. This acceleration of the TCA could possibly enhance the conversion of glutamate to α-ketoglutarate as well as the subsequent metabolization of the latter compound in order to reduce the α-ketoglutarate pool. Two different systems are available in *P. putida* KT2440 to produce glutamate from α-ketoglutarate: a system based on glutamate dehydrogenase (Gdh) that works under nitrogen non-limiting conditions and a system based on glutamate synthase (GOGAT) and glutamine synthetase (GS) that works under nitrogen limiting conditions [102,103]. All cultivations including the *Control* were performed under dual (carbon, nitrogen) limited conditions. Therefore, it is not surprising that the induction of glutamate metabolism we observed involved the GOGAT/GS system and not the Gdh system.

A whole operon encoding proteins from the arginine deiminase (ADI) pathway was found to be repressed in the *Pressure Oxygen* condition (PP_0997-PP_1002; Table 3). This pathway consists of three enzymes: the arginine deiminase which degrades arginine into citrulline and ammonia (PP_1001), the catabolic ornithine carbamoyltransferase which converts citrulline and phosphate into ornithine and carbamoyl phosphate (PP_1000) and the carbamate kinase which produces ATP, ammonia and CO_2_ from carbamoyl phosphate (PP_0999). In addition, the genes encoding these three enzymes are flanked with an arginine/ornithine antiporter (PP_1002) and with a sigma-54 dependent transcriptional regulator/sensory box protein (PP_0997) that is likely to regulate the transcription of the operon. Activation of the ADI pathway was reported to occur via Anr regulation in *P. aeruginosa* and other microorganisms under anaerobic conditions as a mean to generate ATP when terminal electron acceptors such as oxygen and nitrate are scarce [104-107]. The present transcriptome analyses are in agreement with a similar dependency between the transcription of ADI genes, the transcription of *anr* and oxygen availability in *P. putida* KT2440.

## Conclusions

The DNA microarrays and qRT-PCR experiments performed within this work revealed widespread effects of elevated pressure on the transcriptome of *P. putida* KT2440 (Table 2). Growth at 7 bar and elevated DCT was apparently a factor of stress for *P. putida* KT2440, as indicated by the activation of a heat-shock response. In addition, signs of a mild oxidative stress-like response were observed, such as up-regulation of ROS detoxification genes (*ahpC*, glutathione metabolism genes), changes in the composition of terminal oxidases and differential expression of bacterioferritin and siderophore transporters indicating an alteration of iron homeostasis. It should be noted that these stresses could be efficiently fought by the cells, for which no major physiological defects had been detected [2]. The global regulator Anr known for modulating the cellular response to DOT changes was repressed in the *Pressure Oxygen* condition but, unexpectedly, induced in the *Pressure* condition. Thus, it can be concluded that Anr may play an alternative role in the cellular response to elevated pressure and/or DCT. Lastly, the cell envelope appeared to be strongly affected by elevated pressure and/or DCT.

Taken together, the results from the physiological and transcriptomic studies revealed that the variations in pressure, DCT and DOT were important enough to be sensed by the cells and to induce a reorganization of the gene expression pattern while being small enough not to alter significantly the cell physiology.

Furthermore, the data presented here suggest that stimulating the heat-shock and the oxidative-stress responses would be a sensible approach for enhancing the tolerance of *P. putida* against elevated pressure and/or DCT.

## Methods

### Culture conditions and sampling

*P. putida* KT2440 was cultivated in dual (carbon, nitrogen) limited chemostat cultivations in a 16 L high-pressure bioreactor (Bioengineering, Wald, Switzerland) as described previously [2]. The cultivation at 1 bar and DOT = 40% was used as control (*Control*), the one at 7 bar and DOT = 45% to study the effect of elevated pressure (*Pressure*), and the one at 7 bar and DOT = 235% to study the effect of combined elevated pressure and elevated DOT (*Pressure Oxygen*) (Table 1). Three to four culture samples were taken for each cultivation process after steady state was established and at 12 h-intervals. The samples were immediately cooled on ice for 15 min to stop cell growth and RNA degradation. They were then pelleted by centrifugation, incubated 1 h in RNA*later* solution (Ambion, Austin, USA), pelleted again, flash frozen in liquid nitrogen, and stored at -80°C until RNA extraction. Aliquots of about 80 OD-unit (OD_600_ x volume in mL) were prepared. The samples at different time points were not pooled but treated separately and used individually in the four replicate DNA microarrays.

### RNA isolation and purification

RNA isolation and purification were performed according to Yuste *et al.*[108] with minor variations. It involved cell lysis using TRI Reagent® LS (Molecular Research Center Inc., Cincinnati, USA), DNAse treatment with DNase I recombinant (Roche Diagnostics Corporation, Indianapolis, USA), verification of the absence of DNA contamination by polymerase chain reaction (PCR), final RNA purification with RNeasy mini kit (Qiagen, Valencia, USA), and verification of the integrity of the RNA samples by capillary electrophoresis (Agilent 2100 Bioanalyzer, California, USA).

### Hybridization and processing of the microarrays

The hybridization experiments were performed with 2-color DNA microarrays designed for *P. putida* KT2440 [108] and printed by Progenika Biopharma (Vizcaya, Spain). Samples were fluorescently labeled with either Cy3 or Hy5, mixed, and used to hybridize the DNA microarray as reported [108]. Four replicate microarrays were used for each of the two tested conditions (elevated pressure = *Pressure*, and combined elevated pressure and elevated DOT = *Pressure Oxygen*). Microarray data are available in the ArrayExpress database (http://www.ebi.ac.uk/arrayexpress) under accession number E-MEXP-3632.

The results were statistically analyzed using Limma software package [109] in the R environment (http://www.r-project.org). Normalization of the data was performed within arrays using the method *Print-tip loess* and between arrays using the method *Scale*[110]. A linear fit was applied to the four replicate microarrays taking into account the fact that spots were printed in duplicates [111], *i. e.* differential expression was calculated from eight values. The genes were tested for differential expression using the empirical Bayes-moderated *t*-statistics. P-values were adjusted for multiple testing with the Benjamini-Hochberg method to control the false discovery rate [112].

### Data analyses

The large amount of data generated by these experiments (> 5´000 ORFs tested) was analyzed with the following strategy. In a first step, only the genes exhibiting differential expression with a high significance level were considered (adj. P-value < 0.01; Additional file 1: Table S1-S2) in order to identify the most relevant genes and cell functions associated with the response to pressure and DOT increase. Two genes (*ttga* and *aer-1*/PP_2257) were removed from this list and considered as “false positives”: the former belongs to the few extra genes added on the DNA microarray that are absent from *P. putida* KT2440 genome and the latter showed discrepant expression results in qRT-PCR experiments, possibly because of a lack of specificity of its DNA microarray probe. The significantly differentially expressed genes were sorted into role categories and sub-categories according to the Comprehensive Microbial Resource (CMR) [37]. Fisher’s exact tests were performed in the R environment to determine whether (sub-) categories were over-represented in the *Pressure* condition and in the *Pressure Oxygen* condition compared to the genome distribution. The tests were performed with the alternative “greater”, and the significance threshold was set to a P-value of 0.15 in order to obtain a sensible number of relevant functions. In a second step, the expression of specific genes with an adjusted P-value slightly below the significance threshold of 0.01 but with a fold change higher than 1.5 was considered as well (Table 3). Information about the genes was collected from literature search, from the *Pseudomonas* Genome Database publication [113], and from the Kyoto Encyclopedia of Genes and Genomes (KEGG) [101].

### Real Time quantitative Reverse Transcription PCR (qRT-PCR) assay

Reverse transcription reactions for the synthesis of total cDNA were carried out with 4 μg of RNA, 0.5 mM dNTPs, 200 U of SuperScript II Reverse Transcriptase (Invitrogen, Carlsbad, USA) and 2.5 μM of random hexamers as primers in the buffer recommended by the manufacturer. Samples were initially heated at 65°C for 5 min, then incubated at 42°C for 2 h, and the amplification was terminated by incubation at 70°C for 15 min. The cDNA obtained was purified using Geneclean Turbo kit (MP Biomedicals, Santa Ana, USA) and its concentration was measured using a NanoPhotometer™ Pearl (Implen, Munich, Germany). Real time PCR was performed using iQ SYBR Green Supermix (Bio-Rad Laboratories, Hercules, USA) with 0.2 μM of each primer in an iQ5 Multicolor Real-Time PCR Detection System (Bio-Rad Laboratories, Hercules, USA). The primers used for each target gene are listed in the Additional file 1. The samples were initially denatured at 95°C for 5 min, followed by 40 cycles of amplification (95°C, 30 s; 60°C, 30 s and 72°C, 30 s). Three biological and two technical replicates were used for each sample. Data were analyzed with the 2^-ΔΔCt^ method [114] using *rpoN* as internal control as its expression is known to be constant throughout the growth curve [108,115]. Moreover, we have observed that the levels of *rpoN* mRNA were similar under the conditions tested (data not shown).

For the analysis of the expression levels of the target gene *aer-1* hydrolysis probe-based real time PCR was performed using probe n° 70 from the Universal ProbeLibrary (Roche, Basel, Switzerland), with 5 ng of purified cDNA as template, iQ™ Supermix (Bio-Rad Laboratories, Hercules, USA) and 1.25 μM of each primer (see Additional file for the sequences) in an iQ5 Multicolor Real-Time PCR Detection System (Bio-Rad Laboratories, Hercules, USA). Samples were initially denatured at 95°C for 10 min, followed by 50 cycles of amplification (95°C, 10 s; 60°C, 30 s). Three biological and two technical replicates were used for each sample. Data was analyzed using the 2^-ΔCt^ method [114].

## Competing interests

The authors declare that they have no competing interests.

## Authors’ contributions

SF carried out the chemostat cultivations for the production of biomass samples together with BH, performed the RNA extraction and purification together with IFE, did the data treatment and interpretation of the DNA microarray experiments with support from IFE and MAP, and drafted the manuscript. PF performed the qRT-PCR experiments. MZ and MAP participated in the design of the study and, together with SP, IFE and PF, helped with the manuscript preparation. All authors read and approved the final manuscript.

## Supplementary Material

Additional file 1: Table S1 Significantly differentially expressed genes under elevated pressure (Pressure) and elevated pressure and DOT (Pressure Oxygen). **Table S2.** Categories and sub-categories significantly over-represented in the lists of significantly differentially expressed genes under elevated pressure. **Table S3.** Categories and sub-categories significantly over-represented in the lists of significantly differentially expressed genes under combined elevated pressure and elevated DOT. **Table S4.** Top genes with the most significant differential expression at elevated pressure (Pressure) and at combined elevated pressure and DOT (Pressure Oxygen). Click here for file
