# Supplementary material for: New insights on the reorganization of gene transcription in Pseudomonas putida KT2440 at elevated pressure
Source: Microb Cell Fact. 2013 Mar 28;12:30. doi: 10.1186/1475-2859-12-30 (PMC3621253; doi:10.1186/1475-2859-12-30)
Supplement: Additional file 1: Table S1 — Significantly differentially expressed genes under elevated pressure (Pressure) and elevated pressure and DOT (Pressure Oxygen). Table S2. Categories and sub-categories significantly over-represented in the lists of significantly differentially expressed genes under elevated pressure. Table S3. Categories and sub-categories significantly over-represented in the lists of significantly differentially expressed genes under combined elevated pressure and elevated DOT. Table S4. Top genes with the most significant differential expression at elevated pressure (Pressure) and at combined elevated pressure and DOT (Pressure Oxygen). [file 1475-2859-12-30-S1.docx]

**Additional File**

**List of the primers used for qRT-PCR**

The primers used for qRT-PCR were for:

*anr*: Fw-anr (5'-CCTGTCGGGCATGGATAC-3')
Rv-anr (5'-TCACACACCGAAGTGGTTTC-3')

PP_0104: Fw-PP0104 (5'-CAGCCAGCGTCACCTTCT-3')
Rv-PP0104 (5'-CCATGATCGAGCTGATACCC-3')

PP_0504: Fw-PP0504 (5'-CTACATGCTCACCGACCACA-3')
Rv-PP0504 (5'-GTGTGCTTGAACGGGGTAG-3')

PP_2258: Fw-PP2258 (5'-CTTGAGTTACCTGAAACGCTTG-3')
Rv-PP2258 (5'-GTCACGCACGAACGACTG-3')

PP_2439: Fw-PP2439 (5'-CAAGATTGTCGAGCTGAACG-3')
Rv-PP2439 (5'-GCAGCCTTGACCTTACGC-3')

PP_3087: Fw-PP3087 (5'-GTGTACGTGGTGGAGCATGA-3')
Rv-PP3087 (5'-TACATCCACCAGCCAGTCG-3')

PP_3788: Fw-PP3788 (5'-GGTCAAGCGCAAGGGTAAG-3')
Rv-PP3788 (5'-GGCATATGAGCGATCAGGA-3')

PP_4255: Fw-PP4255 (5'-TGGGCATGGTAATGTCGAT-3')
Rv-PP4255 (5'- AGGGTCATCATGCCGTTG-3')

PP_4870: Fw-PP4870 (5'-CGAAAGCTGTAGCGGTATCC-3')
Rv-PP4870 (5'- GTCGACAGTGACCGAGCA-3')

*rpoN:* Fw-rpoN (5'-TCGACCCGGAGCTGGATA-3')
Rv-rpoN (5'-CGGCTCGAACTGCTGGAT-3')

**The primers used for** hydrolysis probe-based qRT-PCR were for:

aer Fw-aer1-70 (5'-GGTATTGCCCAGCACAGC-3')
Rv-aer1-70 (5'-ACACGTGTTCCAGTTCGGTA-3')

**Table S1 - Significantly differentially expressed genes under elevated pressure (*Pressure*) and elevated pressure and DOT (*Pressure Oxygen*)**

**Genes with an adjusted p-value (adj.P.Val) lower than 0.01. Status = type of gene (genomic or extra gene such as antibiotic marker or reporter gene), FC = fold change of the fluorescence intensity between the *Control* and the experimental condition (positive values indicate the increase of expression compared to the *Control* while negative values indicate the decrease of expression compared to the *Control*), AveExp = average between the log of the fluorescence intensities of the control and experimental conditions.**

| **ID** | **Name** | ***Pressure*** | | | ***Pressure Oxygen*** | | |
| --- | --- | --- | --- | --- | --- | --- | --- |
|  |  | **FC** | **AveExp** | **adj.P.Val** | **FC** | **AveExpr** | **adj.P.Val** |
| PP_0033 | sugar transferase putative | -1.74 | 10.35 | 1.88E-03 | -2.21 | 10.71 | 9.61E-03 |
| PP_0103 | cytochrome c oxidase subunit II |  |  |  | -1.67 | 12.52 | 2.57E-03 |
| PP_0104 | cytochrome c oxidase subunit I |  |  |  | -1.68 | 10.86 | 5.58E-03 |
| PP_0111 | Sco1/SenC family protein |  |  |  | -1.44 | 11.66 | 8.74E-03 |
| PP_0153 | conserved hypothetical protein |  |  |  | 1.67 | 14.89 | 8.22E-04 |
| PP_0267 | outer membrane ferric siderophore receptor putative |  |  |  | 1.72 | 12.12 | 6.55E-03 |
| PP_0432 | N-acetyl-gamma-glutamyl-phosphate reductase putative |  |  |  | 2.03 | 12.61 | 1.66E-03 |
| PP_0452 | translation elongation factor Tu | 1.44 | 14.79 | 7.74E-03 | -1.67 | 14.21 | 6.55E-03 |
| PP_0483 | excinuclease ABC A subunit |  |  |  |  |  |  |
| PP_0504 | outer membrane protein OprG | 2.54 | 12.18 | 5.83E-04 | -3.38 | 10.77 | 3.06E-04 |
| PP_0508 | conserved hypothetical protein | 2.54 | 10.35 | 8.76E-03 |  |  |  |
| PP_0536 | hypothetical protein |  |  |  | -1.65 | 11.87 | 1.43E-03 |
| PP_0641 | hypothetical protein | 1.52 | 12.75 | 3.57E-03 | 1.5 | 13.4 | 5.98E-03 |
| PP_0806 | surface adhesion protein putative | -1.58 | 10.33 | 6.17E-03 |  |  |  |
| PP_0811 | cyoups2 protein | 1.79 | 11.98 | 1.88E-03 | 1.95 | 12.15 | 7.20E-04 |
| PP_0812 | cytochrome o ubiquinol oxidase subunit II |  |  |  | 1.59 | 11.9 | 3.01E-03 |
| PP_0841 | rrf2 family protein |  |  |  | 1.67 | 11.6 | 2.33E-03 |
| PP_0842 | cysteine desulfurase |  |  |  | 2.5 | 13.19 | 5.70E-05 |
| PP_0843 | iron-binding protein IscU |  |  |  | 2.48 | 12.27 | 1.48E-04 |
| PP_0844 | iron-binding protein IscA |  |  |  | 2.19 | 13.05 | 1.06E-04 |
| PP_0845 | co-chaperone Hsc20 |  |  |  | 2.46 | 11.22 | 7.60E-04 |
| PP_0847 | ferredoxin 2Fe-2S |  |  |  | 1.72 | 11.61 | 2.21E-03 |
| PP_0848 | conserved hypothetical protein |  |  |  | 1.51 | 12.74 | 5.16E-03 |
| PP_0906 | multidrug efflux RND transporter putative | -2.12 | 12.73 | 1.44E-04 | -2.19 | 13.6 | 2.12E-03 |
| PP_0908 | conserved hypothetical protein | -1.49 | 10.84 | 7.78E-03 | -1.68 | 10.79 | 1.84E-03 |
| PP_0916 | transporter LysE family |  |  |  | 1.83 | 13.88 | 1.63E-03 |
| PP_0997 | sigma-54 dependent transcriptional regulator/sensory |  |  |  | -1.88 | 12.4 | 4.69E-04 |
| PP_0999 | carbamate kinase |  |  |  | -1.72 | 11.9 | 1.34E-03 |
| PP_1000 | ornithine carbamoyltransferase catabolic |  |  |  | -1.53 | 11.6 | 9.61E-03 |
| PP_1001 | arginine deiminase |  |  |  | -1.73 | 11.6 | 1.93E-03 |
| **ID** | **Name** | ***Pressure*** | | | ***Pressure Oxygen*** | | |
|  |  | **FC** | **AveExp** | **adj.P.Val** | **FC** | **AveExpr** | **adj.P.Val** |
| PP_1025 | 2-isopropylmalate synthase |  |  |  | 1.49 | 12.15 | 3.90E-03 |
| PP_1055 | type II secretion pathway protein GspN | 1.51 | 10.33 | 3.07E-03 | 1.80 | 11.58 | 1.34E-03 |
| PP_1068 | amino acid ABC transporter ATP-binding protein |  |  |  |  |  |  |
| PP_1076 | glycerol uptake facilitator protein | 2.23 | 10.95 | 3.04E-03 | 2.02 | 10.79 | 4.69E-04 |
| PP_1079 | ornithine carbamoyltransferase |  |  |  | 1.69 | 10.95 | 3.86E-03 |
| PP_1082 | bacterioferritin |  |  |  | -4.20 | 11.97 | 1.13E-05 |
| PP_1083 | bacterioferritin-associated ferredoxin putative |  |  |  | 2.35 | 11.44 | 1.38E-03 |
| PP_1092 | endonuclease III | -1.67 | 13.09 | 2.24E-03 | -1.58 | 13.55 | 4.08E-03 |
| PP_1111 | synthetase putative |  |  |  | 1.44 | 12.24 | 7.41E-03 |
| PP_1126 | hydrolase putative |  |  |  | -1.41 | 11.49 | 9.64E-03 |
| PP_1129 | pyridoxamine-phosphate oxidase |  |  |  | -1.58 | 11.36 | 3.01E-03 |
| PP_1143 | 3-hydroxyisobutyrate dehydrogenase family protein | -1.69 | 10.67 | 9.95E-03 | -1.94 | 11.17 | 1.82E-03 |
| PP_1150 | membrane protein putative |  |  |  | -4.15 | 13.5 | 3.06E-04 |
| PP_1185 | outer membrane protein H1 | 6.62 | 10.94 | 2.89E-04 | 4.74 | 10.2 | 1.07E-04 |
| PP_1194 | hypothetical protein |  |  |  | -1.67 | 10.44 | 7.88E-03 |
| PP_1207 | conserved hypothetical protein | 1.82 | 14.63 | 2.24E-03 |  |  |  |
| PP_1302 | conserved hypothetical protein TIGR00486 | 1.58 | 10.97 | 4.27E-03 | 1.62 | 11.95 | 2.08E-03 |
| PP_1361 | chaperonin 60 kDa |  |  |  | 2.19 | 13.31 | 6.03E-03 |
| PP_1389 | carboxyphosphonoenolpyruvate phosphonomutase putativ | -1.53 | 10.57 | 3.67E-03 |  |  |  |
| PP_1429 | sigma factor algU regulatory protein AlgN | 1.63 | 11.72 | 1.46E-03 |  |  |  |
| PP_1522 | cold shock protein CspA | -1.50 | 14.39 | 9.09E-03 | -1.75 | 15.13 | 3.26E-03 |
| PP_1624 | group II intron-encoding maturase | -1.68 | 14.81 | 1.88E-03 | -1.73 | 15.19 | 1.34E-03 |
| PP_1630 | recX protein | 1.54 | 12.03 | 2.25E-03 |  |  |  |
| PP_1631 | conserved hypothetical protein TIGR00730 | -1.90 | 10.79 | 2.97E-03 |  |  |  |
| PP_1640 | conserved hypothetical protein |  |  |  | -1.49 | 11.49 | 9.35E-03 |
| PP_1659 | conserved hypothetical protein | -1.95 | 10.26 | 1.88E-03 | -2.20 | 10.2 | 6.37E-03 |
| PP_1683 | transcriptional regulator MarR family |  |  |  | 1.96 | 11.11 | 8.14E-04 |
| PP_1684 | transporter putative |  |  |  | 1.50 | 11.5 | 7.53E-03 |
| PP_1686 | glutathione peroxidase |  |  |  | 2.11 | 11.35 | 3.06E-04 |
| PP_1761 | sensory box protein/GGDEF family protein | -1.77 | 11.64 | 1.88E-03 | -1.71 | 11.71 | 2.78E-03 |
| PP_1762 | conserved hypothetical protein |  |  |  | -1.53 | 13.11 | 9.35E-03 |
| PP_1791 | aldolase/synthase putative | 1.70 | 12.13 | 3.04E-03 |  |  |  |
| PP_1863 | transcriptional regulator LysR family | 2.76 | 12.97 | 3.10E-03 |  |  |  |
| PP_1876 | conserved hypothetical protein |  |  |  | -1.54 | 11.53 | 5.17E-03 |
| PP_1981 | NifR3/Smm1 family protein | 2.84 | 13.81 | 1.44E-04 | 2.38 | 13.85 | 2.09E-03 |
| PP_1991 | hypothetical protein |  |  |  | -2.14 | 12.62 | 1.07E-04 |
| PP_2088 | RNA polymerase sigma factor SigX | -1.87 | 12.67 | 1.88E-03 | -1.64 | 12.84 | 5.58E-03 |
| PP_2097 | sensory box protein | -1.49 | 11.17 | 5.01E-03 |  |  |  |
| **ID** | **Name** | ***Pressure*** | | | ***Pressure Oxygen*** | | |
|  |  | **FC** | **AveExp** | **adj.P.Val** | **FC** | **AveExpr** | **adj.P.Val** |
| PP_2099 | conserved hypothetical protein |  |  |  | 1.53 | 12.48 | 5.58E-03 |
| PP_2119 | ABC efflux transporter permease/ATP-binding protein | -2.19 | 13.81 | 4.10E-03 | -2.10 | 14.59 | 1.23E-03 |
| PP_2135 | hypothetical protein | -3.68 | 14.37 | 1.51E-03 | -4.21 | 13.99 | 1.66E-05 |
| PP_2160 | conserved hypothetical protein |  |  |  | -1.59 | 13.37 | 1.76E-03 |
| PP_2294 | hypothetical protein |  |  |  | -1.53 | 13.24 | 5.58E-03 |
| PP_2295 | antirestriction protein putative | -1.45 | 12.53 | 9.09E-03 | -1.51 | 12.88 | 5.36E-03 |
| PP_2324 | phospho-2-dehydro-3-deoxyheptonate aldolase class I |  |  |  | 1.88 | 12.23 | 3.26E-03 |
| PP_2327 | transcriptional regulator CysB |  |  |  | 1.53 | 11.3 | 8.08E-03 |
| PP_2341 | 6-pyruvoyl tetrahydrobiopterin synthase putative |  |  |  | 1.57 | 11.28 | 9.51E-03 |
| PP_2344 | conserved hypothetical protein | 1.76 | 11.34 | 1.88E-03 | 1.73 | 11.34 | 4.35E-04 |
| PP_2356 | phytochrome family protein putative | -1.72 | 11.75 | 4.57E-03 | -1.85 | 12.6 | 1.34E-03 |
| PP_2373 | hypothetical protein | -2.14 | 14.87 | 9.07E-04 | -3.39 | 14.66 | 2.87E-04 |
| PP_2378 | yhgI protein |  |  |  | 2.14 | 12.58 | 1.46E-04 |
| PP_2405 | conserved hypothetical protein | -1.54 | 11.29 | 2.72E-03 |  |  |  |
| PP_2439 | alkyl hydroperoxide reductase C subunit | 1.87 | 10.73 | 3.76E-03 | 3.89 | 11.49 | 2.51E-05 |
| PP_2445 | conserved hypothetical protein | -1.94 | 12.16 | 1.88E-03 | -2.25 | 12.71 | 1.31E-03 |
| PP_2454 | ribose ABC transporter periplasmic ribose-binding protein |  |  |  | 3.80 | 12.07 | 5.38E-05 |
| PP_2475 | transcriptional regulator TetR family | 1.59 | 11.75 | 7.81E-03 | 1.75 | 11.93 | 7.53E-03 |
| PP_2476 | alcohol dehydrogenase zinc-containing | 1.80 | 10.72 | 1.88E-03 | 2.51 | 10.77 | 5.66E-04 |
| PP_2500 | conserved hypothetical protein | 1.55 | 12.1 | 4.49E-03 | 1.48 | 12.57 | 4.56E-03 |
| PP_2643 | methyl-accepting chemotaxis transducer | -6.8 | 12.96 | 2.87E-07 | -15.41 | 12.09 | 8.55E-08 |
| PP_2645 | magnesium-translocating P-type ATPase |  |  |  | -2.38 | 10.05 | 1.31E-03 |
| PP_2691 | hypothetical protein |  |  |  | -1.77 | 9.98 | 8.22E-03 |
| PP_2697 | flavin reductase domain protein | 2.06 | 10.63 | 2.24E-03 |  |  |  |
| PP_2874 | hypothetical protein |  |  |  | -2.08 | 11.11 | 9.60E-04 |
| PP_2875 | hypothetical protein | 1.52 | 13.26 | 8.42E-03 | 0.89 | 13.89 | 4.69E-04 |
| PP_2882 | 2-hydroxychromene-2-carboxylate isomerase putative | 1.68 | 10.79 | 3.83E-03 |  |  |  |
| PP_3012 | hypothetical protein |  |  |  | -2.00 | 10.94 | 1.31E-03 |
| PP_3023 | amino acid efflux protein putative |  |  |  | 1.50 | 12.26 | 6.37E-03 |
| PP_3036 | hypothetical protein | -2.06 | 11.15 | 1.51E-03 | -2.28 | 11.22 | 1.31E-03 |
| PP_3087 | excinuclease ABC A subunit putative | 1.64 | 10.74 | 3.07E-03 | 2.26 | 11.31 | 1.71E-03 |
| PP_3093 | conserved hypothetical protein | 1.64 | 10.3 | 6.12E-03 | 2.40 | 10.49 | 3.56E-03 |
| PP_3094 | hypothetical protein |  |  |  | 2.61 | 10.1 | 3.51E-05 |
| PP_3095 | chaperone-associated ATPase putative |  |  |  | 1.79 | 10.15 | 5.58E-03 |
| PP_3098 | conserved hypothetical protein |  |  |  | 2.27 | 9.94 | 2.31E-04 |
| PP_3100 | conserved hypothetical protein | 2.26 | 11.33 | 2.89E-04 | 2.69 | 11.31 | 9.60E-04 |
| PP_3130 | hypothetical protein | -1.88 | 11.53 | 1.51E-03 | -2.19 | 11.95 | 2.31E-04 |
| PP_3132 | polysaccharide transporter putative |  |  |  | -1.75 | 10.13 | 7.20E-04 |
| **ID** | **Name** | ***Pressure*** | | | ***Pressure Oxygen*** | | |
|  |  | **FC** | **AveExp** | **adj.P.Val** | **FC** | **AveExpr** | **adj.P.Val** |
| PP_3161 | benzoate dioxygenase alpha subunit |  |  |  | 1.56 | 9.93 | 5.87E-03 |
| PP_3293 | conserved hypothetical protein | -1.47 | 10.47 | 4.50E-03 | -1.74 | 10.77 | 6.37E-03 |
| PP_3320 | conserved domain protein | 2.21 | 10.25 | 1.88E-03 |  |  |  |
| PP_3324 | conserved hypothetical protein | 1.94 | 10.15 | 2.24E-03 |  |  |  |
| PP_3402 | hypothetical protein | -1.79 | 11.54 | 3.16E-03 | -1.96 | 11.64 | 7.60E-04 |
| PP_3422 | lytic transglycosylase | -1.74 | 10.64 | 1.88E-03 | -1.98 | 11.01 | 9.88E-04 |
| PP_3439 | transcriptional regulator AraC family | -1.55 | 11.33 | 7.74E-03 | -1.59 | 11.86 | 5.36E-03 |
| PP_3444 | glyoxalase family protein | -1.57 | 10.76 | 4.46E-03 |  |  |  |
| PP_3530 | conserved hypothetical protein |  |  |  | 1.64 | 11.55 | 1.37E-03 |
| PP_3580 | hypothetical protein |  |  |  | -1.46 | 14.19 | 5.94E-03 |
| PP_3610 | hypothetical protein | -1.51 | 11.7 | 7.97E-03 | -1.82 | 11.97 | 7.53E-03 |
| PP_3639 | alkylhydroperoxidase AhpD domain protein | -1.68 | 11.89 | 6.74E-03 | -1.59 | 12.16 | 2.09E-03 |
| PP_3662 | decarboxylase family protein | -1.85 | 11.66 | 4.87E-03 | -1.69 | 11.94 | 3.90E-03 |
| PP_3698 | conserved hypothetical protein | -1.46 | 10.06 | 5.63E-03 | -1.64 | 10.01 | 1.31E-03 |
| PP_3703 | hypothetical protein | -1.49 | 13.34 | 7.97E-03 | -1.71 | 13.69 | 8.87E-04 |
| PP_3708 | hypothetical protein | 1.61 | 12.44 | 3.55E-03 |  |  |  |
| PP_3729 | periplasmic amino acid-binding protein-related protei |  |  |  | 1.42 | 11.16 | 8.72E-03 |
| PP_3742 | glutathione S-transferase family protein |  |  |  | 1.52 | 11.84 | 5.58E-03 |
| PP_3754 | beta-ketothiolase | 1.76 | 12.17 | 1.93E-03 |  |  |  |
| PP_3775 | sarcosine oxidase putative |  |  |  | 1.43 | 13.66 | 6.76E-03 |
| PP_3776 | rarD protein |  |  |  | 1.70 | 11.6 | 3.55E-03 |
| PP_3780 | hypothetical protein | 1.45 | 12.02 | 7.97E-03 | 1.71 | 12.49 | 1.37E-03 |
| PP_3782 | hypothetical protein |  |  |  | 1.91 | 13.92 | 7.60E-04 |
| PP_3783 | conserved hypothetical protein | 1.60 | 14.07 | 2.74E-03 | 1.94 | 14.43 | 2.84E-04 |
| PP_3784 | conserved domain protein |  |  |  | 1.78 | 12.03 | 7.69E-03 |
| PP_3785 | hypothetical protein | 1.64 | 12.37 | 9.95E-03 | 1.86 | 12.36 | 3.16E-03 |
| PP_3786 | aminotransferase |  |  |  | 1.78 | 12.84 | 7.26E-03 |
| PP_3787 | hypothetical protein | 2.10 | 12.34 | 5.30E-03 | 2.35 | 12.73 | 9.15E-04 |
| PP_3788 | non-ribosomal peptide synthetase putative | 2.35 | 11.33 | 3.67E-03 | 2.80 | 11.69 | 4.24E-05 |
| PP_3790 | diaminopimelate epimerase |  |  |  | 1.99 | 10.98 | 1.38E-03 |
| PP_3825 | conserved hypothetical protein |  |  |  | 1.51 | 12.15 | 9.49E-03 |
| PP_3829 | molybdate ABC transporter permease protein |  |  |  | 1.49 | 12.35 | 5.58E-03 |
| PP_3839 | alcohol dehydrogenase zinc-containing |  |  |  | -1.68 | 12.41 | 5.58E-03 |
| PP_4030 | enoyl-CoA hydratase/isomerase family protein |  |  |  | 1.55 | 11.31 | 4.56E-03 |
| PP_4037 | glutamate synthase small subunit putative |  |  |  | -1.90 | 14.06 | 5.58E-03 |
| PP_4038 | dihydroorotate dehydrogenase family protein |  |  |  | -1.76 | 13.7 | 7.53E-03 |
| PP_4045 | conserved hypothetical protein |  |  |  | -1.72 | 13.75 | 3.93E-03 |
| PP_4119 | NADH dehydrogenase I A subunit |  |  |  | -1.65 | 14.32 | 3.62E-03 |
| PP_4179 | heat shock protein HtpG | 1.64 | 11.33 | 9.95E-03 |  |  |  |
| **ID** | **Name** | ***Pressure*** | | | ***Pressure Oxygen*** | | |
|  |  | **FC** | **AveExp** | **adj.P.Val** | **FC** | **AveExpr** | **adj.P.Val** |
| PP_4202 | electron transfer flavoprotein beta subunit | 1.43 | 14.36 | 6.25E-03 | 1.82 | 14.85 | 1.37E-03 |
| PP_4251 | cytochrome c oxidase cbb3-type subunit II |  |  |  | -2.23 | 9.73 | 1.95E-03 |
| PP_4255 | cytochrome c oxidase cbb3-type subunit I |  |  |  | -1.54 | 12.05 | 7.36E-03 |
| PP_4261 | cation-transporting P-type ATPase |  |  |  | -1.76 | 11.69 | 1.91E-03 |
| PP_4314 | conserved hypothetical protein |  |  |  | 2.77 | 11.83 | 7.76E-03 |
| PP_4362 | conserved hypothetical protein |  |  |  | -1.52 | 11.65 | 9.02E-03 |
| PP_4384 | flagellar L-ring protein precursor FlgH | -1.49 | 11.23 | 5.63E-03 |  |  |  |
| PP_4405 | sensory box protein | 2.04 | 11.16 | 7.97E-03 |  |  |  |
| PP_4454 | opine ABC transporter permease protein putative | 1.94 | 11.28 | 2.56E-03 |  |  |  |
| PP_4465 | porin putative | 2.32 | 10.29 | 1.88E-03 |  |  |  |
| PP_4503 | DNA-binding response regulator | 1.56 | 12.36 | 2.30E-03 |  |  |  |
| PP_4508 | transcriptional regulator AraC family |  |  |  | -1.54 | 11.9 | 3.56E-03 |
| PP_4573 | ATPase AAA family | 1.89 | 11.68 | 1.93E-03 |  |  |  |
| PP_4592 | membrane protein putative | -1.74 | 14.16 | 3.10E-03 | -2.21 | 14.02 | 1.93E-03 |
| PP_4594 | cystathionine gamma-synthase putative | -1.69 | 15.27 | 1.88E-03 |  |  |  |
| PP_4617 | leucine dehydrogenase | -1.76 | 11.28 | 1.93E-03 | -1.62 | 11.59 | 3.01E-03 |
| PP_4624 | hydrolase alpha/beta fold family | -1.51 | 10.61 | 8.04E-03 |  |  |  |
| PP_4648 | nucleotide methyltransferase putative | -1.43 | 11.31 | 8.46E-03 |  |  |  |
| PP_4715 | triosephosphate isomerase | -1.58 | 11.8 | 3.07E-03 |  |  |  |
| PP_4728 | heat shock protein GrpE |  |  |  | 1.72 | 11.83 | 7.19E-03 |
| PP_4794 | leucyl-tRNA synthetase | -1.92 | 12.08 | 3.41E-04 | -1.73 | 12.2 | 7.53E-03 |
| PP_4841 | branched-chain amino acid ABC transporter periplasmi | 1.95 | 10.6 | 8.52E-03 |  |  |  |
| PP_4842 | branched-chain amino acid ABC transporter permease p | 3.08 | 12.03 | 4.93E-03 |  |  |  |
| PP_4867 | branched-chain amino acid ABC transporter periplasmi | 1.79 | 12.5 | 3.34E-03 |  |  |  |
| PP_4870 | azurin | 2.22 | 12.94 | 1.88E-03 |  |  |  |
| PP_4897 | N-acetylmuramoyl-L-alanine amidase | 1.67 | 12.15 | 3.55E-03 |  |  |  |
| PP_4900 | iron-sulfur cluster-binding protein putative | -1.44 | 10.77 | 4.46E-03 |  |  |  |
| PP_4922 | thiamin biosynthesis protein ThiC |  |  |  | 1.77 | 10.92 | 3.26E-03 |
| PP_4958 | conserved hypothetical protein | -1.51 | 11.91 | 8.42E-03 | -1.68 | 12.45 | 8.08E-03 |
| PP_5073 | conserved hypothetical protein TIGR00156 | 5.69 | 11.14 | 1.88E-03 | 5.96 | 11.42 | 8.08E-06 |
| PP_5108 | RNA polymerase sigma-32 factor | 1.5 | 13.26 | 3.55E-03 | 1.56 | 13.9 | 8.74E-03 |
| PP_5231 | hydrolase haloacid dehalogenase-like family | -1.6 | 14.44 | 6.12E-03 |  |  |  |
| PP_5233 | ammonium transporter | 1.87 | 14.9 | 8.04E-03 |  |  |  |
| PP_5301 | DNA-directed RNA polymerase omega subunit |  |  |  | 1.60 | 12.17 | 1.98E-03 |
| PP_5304 | lipoprotein putative |  |  |  | 1.60 | 10.93 | 3.01E-03 |
| PP_5307 | ferric siderophore transport system inner membrane protein |  |  |  | 2.30 | 10.96 | 1.76E-03 |
| **ID** | **Name** | ***Pressure*** | | | ***Pressure Oxygen*** | | |
|  |  | **FC** | **AveExp** | **adj.P.Val** | **FC** | **AveExpr** | **adj.P.Val** |
| PP_5324 | response regulator | -1.53 | 10.18 | 4.27E-03 | -1.47 | 10.58 | 9.23E-03 |
| PP_5347 | acetyl-CoA carboxylase biotin carboxylase | -2.23 | 11.16 | 1.88E-03 | -2.41 | 11.09 | 5.58E-03 |
| PP_5389 | hypothetical protein |  |  |  | -4.03 | 10.77 | 1.13E-05 |
| PP_5390 | hypothetical protein | 1.95 | 12.27 | 4.46E-03 | -7.22 | 10.64 | 6.94E-06 |
| PP_5391 | hypothetical protein |  |  |  | -6.54 | 11.62 | 1.46E-04 |
| PP_5392 | conserved hypothetical protein |  |  |  | -4.13 | 12.02 | 7.11E-04 |

**Table S2 -** **Categories and sub-categories significantly over-represented in the lists of significantly differentially expressed genes under elevated pressure.**

| (Sub-)category | genes | genes [%] | P-val^#^ |
| --- | --- | --- | --- |
| ***Hypothetical proteins*** | 32 | 2.02 | 0.002 |
| Conserved hyp. proteins | 16 | 1.67 | 0.074 |
| Hypothetical proteins | 15 | 2.53 | 0.096 |
| ***Regulatory functions **** |  |  |  |
| Small molecule interactions | 4 | 8.70 | 0.008 |
| ***Transcription **** |  |  |  |
| Transcription factors | 2 | 6.67 | 0.097 |

^#^ P-value < 0.15

* Category not significantly over-represented but containing at least one sub-category being over-represented.

**Table S3 - Categories and sub-categories significantly over-represented in the lists of significantly differentially expressed genes under combined elevated pressure and elevated DOT.**

| (Sub-)***category*** | genes [-] | genes [%] | P-val^#^ |
| --- | --- | --- | --- |
| ***Amino acid biosynthesis*** | ***6*** | ***4.65*** | ***0.122*** |
| Glutamate family | 3 | 9.09 | 0.054 |
| ***Biosynth. of cofactors, prosthetic gps, and carriers*** * |  |  |  |
| Glutathione and analogs | 1 | 16.67 | 0.147 |
| Other | 3 | 20.00 | 0.006 |
| ***Cellular processes*** * |  |  |  |
| Adaptations to atypical conditions | 3 | 6.25 | 0.130 |
| ***Energy metabolism*** | ***18*** | ***3.72*** | ***0.081*** |
| Amino acids and amines | 6 | 7.14 | 0.022 |
| Electron transport | 9 | 7.76 | 0.003 |
| ***Hypothetical proteins*** | ***52*** | ***3.29*** | ***0.034*** |
| Hypothetical proteins | 25 | 4.22 | 0.011 |
| ***Protein fate*** * |  |  |  |
| Protein folding and stabilization | 3 | 6.38 | 0.124 |
| Other | 1 | 25.00 | 0.101 |
| ***Transcription*** * |  |  |  |
| DNA-dependent RNA polymerase | 1 | 20.00 | 0.124 |
| ***Unknown function*** | 18 | 3.47 | 0.130 |
| General | 12 | 4.80 | 0.030 |

^#^ P-value < 0.15

* Category not significantly over-represented but containing at least one sub-category being over-represented.

**Table S4 - Top genes with the most significant differential expression at elevated pressure (*Pressure*) and at combined elevated pressure and DOT (*Pressure Oxygen*).**

| ID | Name | Gene | FC * | Adj. p-value |
| --- | --- | --- | --- | --- |
| ***Pressure (adj. p-value ≤ 0.001)*** | | | | |
| PP_2643 ^✝^ | methyl-accepting chemotaxis transducer |  | -6.80 | 2.9 E-07 |
| PP_0906 | multidrug efflux RND transporter putative |  | -2.12 | 1.4 E-04 |
| PP_1981 | NifR3/Smm1 family protein |  | +2.84 | 1.4 E-04 |
| PP_1185 ^✝^ | outer membrane protein H1 | *oprH* | +6.62 | 2.9 E-04 |
| PP_3100 | conserved hypothetical protein (Type VI SS) |  | +2.26 | 2.9 E-04 |
| PP_4794 | leucyl-tRNA synthetase | *leuS* | -1.92 | 3.4 E-04 |
| PP_0504 | outer membrane protein OprG | *oprG* | +2.54 | 5.8 E-04 |
| PP_2373 | hypothetical protein |  | -2.14 | 9.1 E-04 |
| ***Pressure Oxygen (adj. p-value ≤ 0.0001)*** | | | | |
| PP_2643 ^✝^ | methyl-accepting chemotaxis transducer |  | -15.41 | 8.5 E-08 |
| PP_5390 | hypothetical protein |  | -7.22 | 6.9 E-06 |
| PP_5073 | conserved hypothetical protein |  | +5.96 | 8.1 E-06 |
| PP_1082 | bacterioferritin | *bfr* | -4.20 | 1.1 E-05 |
| PP_5389 | hypothetical protein |  | -4.03 | 1.1 E-05 |
| PP_2135 | hypothetical protein |  | -4.21 | 1.7 E-05 |
| PP_2439 | alkyl hydroperoxide reductase C subunit | *ahpC* | +3.89 | 2.5 E-05 |
| PP_3094 | hypothetical protein (Type VI SS) |  | +2.61 | 3.5 E-05 |
| PP_3788 | non-ribosomal peptide synthetase putative |  | +2.80 | 4.2 E-05 |
| PP_2454 | ribose ABC transporter periplasmic ribose-binding pr. | *rbsB* | +3.80 | 5.4 E-05 |
| PP_0842 | cysteine desulfurase | *iscS* | +2.50 | 5.7 E-05 |
| PP_0844 | iron-binding protein IscA | *iscA* | +2.19 | 1.1 E-04 |
| PP_1185 ^✝^ | outer membrane protein H1 | *oprH* | +4.74 | 1.1 E-04 |
| PP_1991 | hypothetical protein |  | -2.14 | 1.1 E-04 |

* **Fold change of the fluorescence intensity between the *Control* and the experimental condition (positive values indicate the increase of expression compared to the *Control* while negative values indicate the decrease of expression compared to the *Control*)**
^✝^ Top genes differentially expressed both for *Pressure* and for *Pressure Oxygen*.
